# *Polystemma cualense* (Apocynaceae), a new species from western Mexico

**DOI:** 10.3897/phytokeys.276.176723

**Published:** 2026-06-18

**Authors:** Cristóbal Daniel Sánchez-Sánchez, Carla Sofía Islas-Hernández, Adam W. Black

**Affiliations:** 1 Departamento de Botánica, Jardín Botánico de Vallarta, Jalisco, Mexico Departamento de Biología Comparada, Laboratorio de Plantas Vasculares, Facultad de Ciencias, Universidad Nacional Autónoma de México Mexico City Mexico; 2 Departamento de Biología Comparada, Laboratorio de Plantas Vasculares, Facultad de Ciencias, Universidad Nacional Autónoma de México, Mexico City, Mexico Departamento de Botánica, Jardín Botánico de Vallarta Jalisco Mexico; 3 Bartlett Tree Research Laboratories and Arboretum, Charlotte, North Carolina, USA Bartlett Tree Research Laboratories and Arboretum Charlotte United States of America

**Keywords:** Asclepiadoideae, high diversity, Jalisco, Sierra El Cuale

## Abstract

A new species of *Polystemma* is described from the Sierra El Cuale, Jalisco, Mexico. *Polystemma cualense* is distinguished from its congeners, particularly *P. horconesense*, by a gynostegial corona higher than the gynostegium, longer external corona lobes, and a black gynostegium. Morphological data were obtained by examining herbarium specimens, consulting digital images from iNaturalist, and reviewing specialized literature. A preliminary conservation assessment based on IUCN criteria indicates that it is a Critically Endangered (CR) species due to its restricted area of occupancy and threats from deforestation, agriculture, and livestock grazing. The discovery of *P. cualense* increases the known diversity of *Polystemma* in Jalisco and highlights the floristic importance of the Sierra El Cuale.

## Introduction

The genus *Polystemma* Decaisne belongs to the subtribe Gonolobinae of Apocynaceae and is distributed from northern Mexico to Costa Rica in Central America (Morillo 2023; Alvarado-Cárdenas et al. 2024a; González-Martínez et al. 2024). It is characterized by its white glandular trichomes that become whitish-crystalline toward maturity, corolla lobes with dense pubescence on the abaxial side, a double gynostegial corona, and smooth, mottled, or longitudinally striated follicles (Morillo 2023; Cervantes-Meza et al. 2024). Phylogenetic studies based on molecular data recover *Polystemma* as a monophyletic group nested within the paraphyletic genus *Matelea* Aublet (González-Martínez et al. 2024). In the last 5 years, botanical exploration in western Mexico has led to the discovery of seven new species of *Polystemma* (*P. atreyui* Pío-León, L.O. Alvarado & S. Islas, *P. fishbeiniana* V.W. Steinm. & W.D. Stevens, *P. galindoi* Pío-León, L.O. Alvarado & S. Islas, *P. horconesense* C.D. Sánchéz, L.O. Alvarado & S. Islas, *P. leopardum* L.O. Alvarado, García-Mend., D. Sandoval & Lozada-Pérez, *P. margaritadelacerdae* J. Martínez-Ramírez, L.O. Alvarado & Ocampo, and *P. stevensii* G.M. Hern-Barón, Trujillo-Juaréz & V.W. Steinm.), and many species have been transferred from *Matelea* and *Gonolobus*. Currently, at least 28 species are recognized in this genus (Steinmann and Stevens 2022; Hernández-Barón et al. 2023; Alvarado-Cárdenas et al. 2024a, 2024b; Ocampo et al. 2025). There has been remarkable progress in the study of Jalisco’s flora, but areas requiring further collecting efforts have also been identified (Gudiño-Cano et. al 2024). An example of this is Sierra El Cuale, where plants with characters that place them in *Polystemma* have recently been detected; however, they have not been identified as any of the species recognized to date and are therefore proposed here as a new species to science.

## Materials and methods

During botanical expeditions to Sierra El Cuale, Jalisco, carried out in 2024 as part of an oak conservation project, a specimen of *Polystemma* was collected and compared with specimens deposited in different national herbaria (FCME, CIIDIR, MEXU; abbreviations *sensu*Thiers 2024) as well as with observations from the iNaturalist platform (2025). Specialized literature on the genus was also reviewed (Alvarado-Cárdenas et al. 2024a, 2024b). Measurements of leaves, inflorescences, and flowers were obtained with a digital caliper, and details of floral structures and trichomes were obtained using a Nikon stereomicroscope (C-Leds, SMZ445, Tokyo, Japan). The description of the leaves was based on Hickey (1973), the indument on Harris and Harris (1994), and the corona on Alvarado-Cárdenas et al. (2024a, 2024b).

The proposal of the new taxon as an explanatory hypothesis follows Alvarado-Cárdenas et al. (2024c), using the cohesive species concept of Templeton (1989) and applying the phenotypic restriction factor to distinguish it from the other species hypotheses.

The specimen coordinates were used to calculate the area of occupancy (AOO), using 2 × 2 km cells with the GeoCAT tool (Bachman et al. 2011) and applying the IUCN criteria (IUCN 2024) to propose a preliminary conservation assessment category.

## Taxonomic treatment

### Polystemma cualense

Taxon classificationPlantaeActiniariaActiniidae

C.D.Sánchez, S.Islas & A.Black sp. nov.

E1B3F0AE-026E-5576-AF8B-AEBD9E687644

urn:lsid:ipni.org:names:77381688-1

Fig. 1

#### Type.

**Mexico** • Jalisco, ejido El Cuale, Talpa de Allende municipality, Los Lobos trail to El Cuale, 20°27'50.3"N, 105°05'39.7"W, 1,030 m, 24 Aug 2024, *C.D.Sánchez, A. Black, J. Reyes and M. Peinado 1701* (Holotype: MEXU!; • Isotypes: PV!).

**Figure 1.**
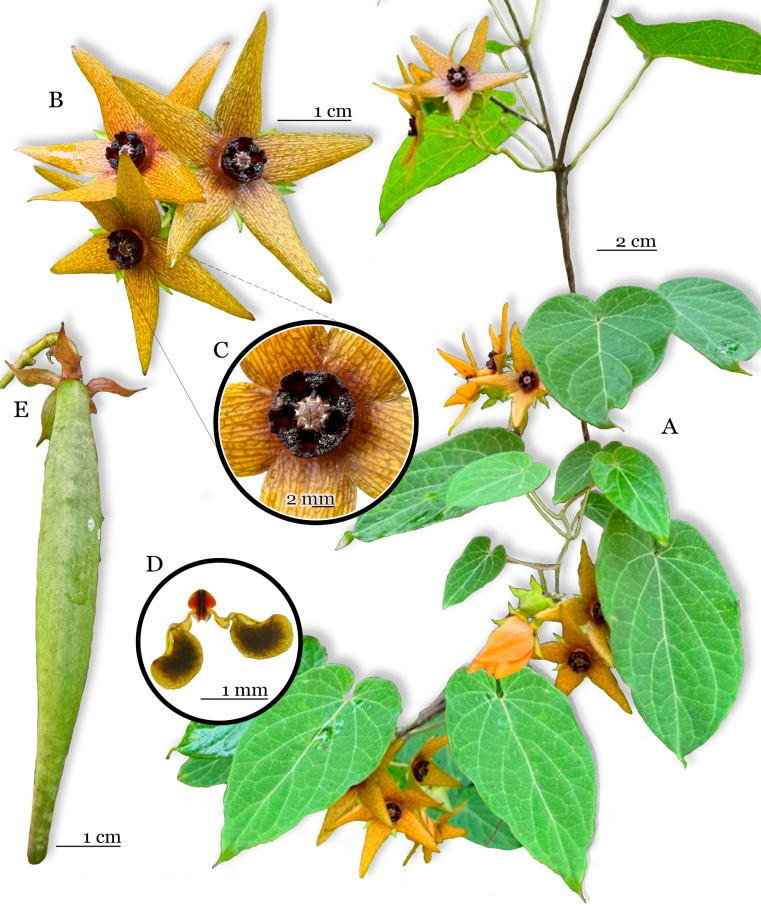
*Polystemma cualense*. **A**. Complete plant; **B**. Inflorescence; **C**. Detail of gynostegium; **D**. Pollinarium. **E**. Immature follicle with sepal remnants.

#### Diagnosis.

*Polystemma cualense* resembles *P. horconesense* in having flowers similar in size and shape. *Polystemma cualense* differs in having a gynostegial corona higher than the gynostegium (vs. gynostegial corona the same height as the gynostegium), external gynostegial corona lobes 3–4 mm length (vs. 0.5–0.6 mm length) and gynostegium black (vs. gynostegium green-yellowish).

#### Description.

Perennial climbing plants. Stems cylindrical, mixed indumentum of long simple trichomes ca. 0.5 mm long oriented along two lines on the sides of the stem, simple short trichomes ca. 0.1 mm long, and glandular trichomes ca. 0.2 mm long, the latter more dense in the nodes. Leaves petiolate, petioles 2.3–5.1 cm long, lamina elliptic-ovate, 5.7–8.8 x 2.8–5.4 cm, base cordate, lobes 6.7–10.5 x 11.3–19.3 mm, apex mucronate, 5–11 mm long, margins entire, ciliate; adaxially with simple trichomes upon the veins, abaxially with glandular trichomes upon the veins; colleters 4–6, at the union of the leaf blade with the petiole, conical to spheroid, 0.3–0.6 mm long, yellowish-brown to dark brown; 3–4 pairs of secondary veins. Inflorescences cymose-umbelliform, one per node, extra-axillary, with 4–6 flowers; peduncles 2.2–3.5 mm long, with mixed indumentum of short simple trichomes and glandular trichomes; bracts two per flower 4.6–5.7 x 1.0–2.0 mm, ovate-lanceolate, with long simple trichomes on the margins. Flowers pentamerous, pedicels 6.5–11.2 mm long with mixed indumentum of long simple trichomes, short simple trichomes, and glandular trichomes; calyx with sepals fused at the base, tube ca. 2.5 mm long, lobes 7.2–10.4 mm x 2.6–3.9 mm, lanceolate, adaxially and abaxially glabrate, margins ciliate, colleter one, ca. 0.4 x 0.3 mm, at the base of the sepals, alternating with them, ovate, yellow; corolla rotate, 2.5–3.5 cm in diameter, deeply lobulate, yellowish-green to yellowish-brown with darker brown reticulum, aestivation valvate in the flower buds, tube 3.4–4.3 x 4.8–6.3 mm, glabrescent, limb 2.9–3.4 mm long, lobes 1.0–1.6 cm x 3.7–5.4 mm, lanceolate, apex acute, abaxially and adaxially glabrous; gynostegial corona higher than the gynostegium, formed by five lobes 2.7–3.3 mm long, ca. 7.4 mm in diameter, black, fused at the base forming an uninterrupted ring, external corona cyathiform, with interstaminal lobes 3–4 x 3.4 mm, laminar, internal corona verrucose, with margins crenulate to dentate, gynostegium 7.6–8.0 mm in diameter, stipitate, stipite ca. 0.5 mm long; gynoceium bicarpellary, apocarpous, glabrous, ca. 3.0 mm long, stylar head flat, ca. 2.5 mm in diameter; androecium with anthers horizontal, ca. 0.6 x 1.0 mm, terminal appendages of anthers suborbicular, ca. 1.5 mm; pollinaria with corpuscles 0.25–0.34 x 0.28–0.28 mm, sagittate, translator 0.24–0.38 mm long, with winged appendages, pollinia 0.52–0.63 x 0.50–0.59 mm, reniform. Immature follicles 9–10.1 x 1–1.1 cm; seeds not seen.

#### Distribution and habitat.

**Figure 2.**
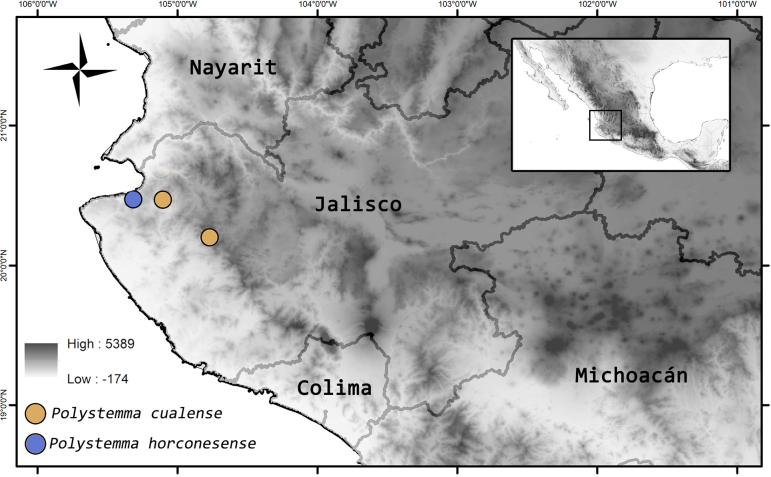
Distribution of *Polystemma cualense* and *Polystemma horconesense*.

*Polystemma cualense* is endemic to the state of Jalisco, growing in ruderal vegetation and relict gallery forest near crop areas in the Talpa de Allende municipality at 1,030–1,300 m altitude (Fig. 2). It is associated with *Mimosa albida* Humb. & Bonpl. ex Willd. (Fabaceae), *Hamelia xorullensis* Kunth (Rubiaceae), *Melampodium perfoliatum* (Cav.) Kunth (Asteraceae), *Milleria quinqueflora* L. (Asteraceae), *Adiantum* sp. (Pteridaceae), naturalized *Bauhinia variegata* L. (Fabaceae), and *Lantana camara* L. (Verbenaceae).

#### Phenology.

Flowering occurs from late July through early September, and mature fruits have been observed in September but have not been collected.

#### Conservation status.

This species is known only from two populations in Talpa de Allende, Jalisco, with an AOO of 8 km^2^ (B2). In 2025, the only three individuals at the type locality were lost to land clearing (A1, C1, and C2), and the remaining habitats are threatened by deforestation, livestock grazing, and agriculture. Despite the potential for undiscovered populations, a Critically Endangered (CR) status (B1+B2) is recommended to facilitate urgent conservation.

#### Etymology.

The name refers to Sierra El Cuale, as the localities of this new species are situated along the margin of this geological formation. This discovery adds to the list of species named in honor of this enigmatic mountain range, whose forests are threatened by land-use change, illegal logging, mining, and fires.

#### iNaturalist records.

Mexico: Jalisco, municipality: Talpa de Allende, 20.2114601133, -104.7813415525, 1070 m, 31 Aug 2019, pcarreyes 90622325 (https://mexico.inaturalist.org/observations/97517086).

#### Taxonomic remarks.

**Figure 3.**
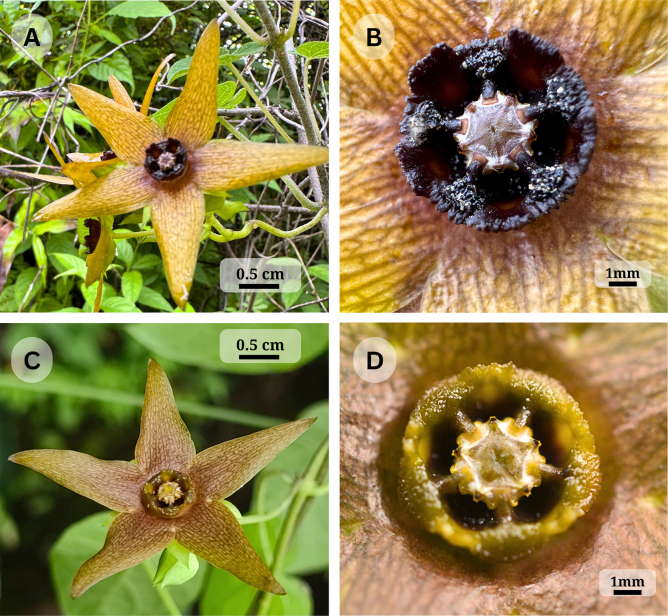
Comparison of the flowers and gynostegia of *Polystemma cualense* (**A**. Flower; **B**. Gynostegium) and *Polystemma horconesense* (**C**. Flower; **D**. Gynostegium).

Exploratory work across the country has led to the description of new species of the genus *Polystemma* in recent years (Alvarado-Cárdenas et al. 2024b). This species shares similarities with *P. horconesense*, as both have flowers of similar corolla size and shape (Fig. 3). *Polystemma cualense* has umbellate-cymose inflorescences (vs. monochasial inflorescences in *P. horconesense*), a single colleter at the base of the sepals (vs. three), a rotate corolla (vs. a campanulate-rotate corolla), yellow flowers (vs. brown or pale greenish flowers with violet hues), and a gynostegial corona that extends beyond the gynostegium (vs. a gynostegial corona of the same height as the gynostegium) (Alvarado-Cárdenas et al. 2024a). These phenotypic differences support the two taxa as hypotheses of distinct species.

*Polystemma* distribution is concentrated primarily on the Atlantic slope, with the western part of the country being an area of high diversity. The Jalisco region is distinguished by its remarkable endemism and recent additions to the state’s flora (Gudiño-Cano et al. 2024), a diversity closely tied to strong environmental heterogeneity along elevation gradients. *Polystemma cualense* occurs above 1,000 m in temperate subhumid conditions, whereas *P. horconesense* is restricted to elevations below 400 m in warm subhumid climates.

This new taxon increases the number of *Polystemma* species in Jalisco to 10, which places it among the richest states in this genus, along with Oaxaca (10 species), Guerrero (eight species), and Michoacán (seven species).

## Supplementary Material

XML Treatment for Polystemma cualense

## Additional information

### Artificial Intelligence (AI) use

The authors accept full responsibility for the content of the manuscript, including the disclosure of any use of AI.

No AI tools were used in the preparation of this manuscript.

### Author ORCIDs

C.D. Sánchez-Sánchez https://orcid.org/0000-0002-3325-8724

C.S. Islas-Hernández https://orcid.org/0000-0003-4496-3127

A.W. Black https://orcid.org/0009-0007-1875-933X

### Data availability

All of the data that support the findings of this study are available in the main text.
